# Internet-Based Social Activities and Cognitive Functioning 2 Years Later Among Middle-Aged and Older Adults: Prospective Cohort Study

**DOI:** 10.2196/63907

**Published:** 2024-12-10

**Authors:** Sangha Jeon, Susan Turk Charles

**Affiliations:** 1 Department of Psychological Science, University of California, 214 Pereira Dr, Irvine, CA, 92617, United States, 1 949-824-6803

**Keywords:** online social interaction, cognitive health, age differences, Health and Retirement Study, social activity, internet use, isolation

## Abstract

**Background:**

A number of studies document the benefits of face-to-face social interactions for cognitive functioning among middle-aged and older adults. Social activities in virtual worlds may confer similar if not enhanced cognitive benefits as face-to-face social activities, given that virtual interactions require the additional cognitive tasks of learning and navigating communicative tools and technology platforms. Yet, few studies have examined whether social activities in internet-based settings may have synergistic effects on cognitive functioning beyond those of face-to-face interactions.

**Objective:**

This study examined whether internet-based social activity participation is associated with concurrent and later cognitive functioning, after adjusting for face-to-face social activity participation and sociodemographic covariates.

**Methods:**

For cross-sectional analyses, we included 3650 adults aged 50 years and older who completed questions in the 2020 Health and Retirement Study about social activity participation, including specific internet-based social activities such as emailing or accessing social networks. Cognitive functioning was measured using the standardized cognitive tasks assessing working memory, episodic memory, and attention and processing speed. The longitudinal analyses included the 2034 participants who also completed follow-up cognitive assessments in 2022.

**Results:**

Our results revealed that those with higher levels of internet-based social activity participation had higher levels of concurrent cognitive functioning than those with low levels of internet-based social activity participation, after adjusting for demographic and health-related factors and face-to-face social activity participation (*b*=0.44, SE 0.07; *P*<.001). More internet-based social activity participation also predicted better cognitive functioning 2 years later, even when adjusting for baseline cognitive functioning and other covariates (*b*=0.35, SE 0.09; *P*<.001).

**Conclusions:**

Our findings suggest that greater engagement in internet-based social activities is associated with higher levels of concurrent cognitive functioning and slower cognitive decline in middle-aged and older adults.

## Introduction

Social activity participation is strongly tied to multiple aspects of health and well-being in later life, including psychological well-being, depressive symptoms, physical health and functional limitations, and cognitive health [1-5]. Emerging research focusing on the cognitive benefits of social activity participation documents different aspects of social activity participation that are related to cognitive functioning, such as frequency (how often an individual engages in social activities) and variety (how many different types of activities an individual engages in social activities) [6]. For example, those who participate in social activities more frequently are likely to have enhanced concurrent cognitive functioning, slower age-related cognitive decline, and reduced dementia risk [7,8]. In addition to frequency of engagement, those who engage in a greater number of social activities show better later cognitive functioning than their socially inactive peers [6]. These social activities increasingly involve virtual interactions such as texting, video chats, or the use of other social media to stay connected [9]. Nevertheless, most research has focused predominantly on the benefits of face-to-face social activity participation on cognitive functioning [8]. This study investigated how engagement in a variety of internet-based social activities relates to cognitive functioning, especially for adults aged 50 years and above.

Communicating with others in virtual worlds can offer similar cognitive health benefits as face-to-face social activities [10]. For example, internet-based social activities such as posting status updates or liking others’ updates on Facebook are associated with high levels of cognitive functioning in adults aged over 55 years [11]. Emailing and texting, also forms of internet-based social connection, are related to a lower likelihood of cognitive impairment over 5 years in adults aged over 65 years [12]. Beyond engaging in any internet-based social activities, engagement in different types of internet-based social activities may also have cognitive benefits.

A variety of internet-based social activities may provide greater opportunities to learn novel information than engaging in fewer activities; moreover, it is related to greater hippocampal volume [13]. Novel information is also related to hippocampal neurogenesis and reduced age-related neural apoptosis, which are associated with cognitive functioning that relies on more than a single source of communication [14,15]. In addition, individuals can also share thoughts or feelings in a variety of virtual communities [16], which engage cognitive processes such as problem-solving and perspective-taking [17].

Unique aspects of internet-based social activities may boost the cognitive benefits of engaging in a variety of such activities. First, different types of internet-based social activities may require the use of various interactive tools such as internet searching or instant messaging. These different techniques and activities may be cognitively stimulating because they involve learning and memory [18]. Even simple internet searches are a neural exercise for middle-aged and older adults, activating multiple brain regions related to decision-making and complex reasoning [19]. Furthermore, content acquired through internet-based social activities is delivered in multiple formats such as sound, pictures, or videos [20]. Studies have demonstrated that the simultaneous presentation of information in visual and auditory modalities is associated with enhanced learning performance [21]. Moreover, each internet-based social activity may have its own function, offering an array of uses. For example, blogs or Facebook are used for information sharing, while Snapchat or Instagram is used for self-expression and self-documentation [22-24]. These findings about the use of different modalities and functions across various platforms suggest that internet-based social activity participation may be associated with better cognitive functioning.

Internet-based social activities may also benefit specific cognitive domains. Although research on the association between internet-based social activity participation and subdomains of cognitive functioning is still emerging, some studies provide initial evidence that benefits may particularly be related to memory. For example, internet use is linked to improved episodic memory, potentially through increased social contact [25]. Concurrent engagement in offline and internet-based social activities is also related to high levels of episodic memory [26]. This may be explained by people recalling previous social interactions and past experiences as well as by the processing and storing of new information, all of which are linked to episodic memory [27,28]. On the other hand, the relationship between internet-based social activity participation and executive functioning is more complex, as executive functioning encompasses a broad range of cognitive control processes such as reasoning, problem-solving, and attentional control [29]. Some studies suggest that learning to engage in internet-based social activities on laptop applications can enhance inhibition, but its relationship with working memory and attention and processing speed is less consistent [30]. Facebook users also exhibit higher scores in attention or inhibition, but not in working memory, than nonusers [11].

Studying technology use in the social domain for older adults may be particularly relevant because people are increasingly motivated to use technology for social motives as they age. According to socioemotional selectivity theory [31], people increasingly prioritize socioemotional goals with age. As a result, people are even more motivated to connect with others such as friends or grandchildren as they grow older, and thus, they are more likely to engage in internet-based platforms for socioemotional goals as opposed to nonsocial goals. While young adults may engage in internet-based social activities for various purposes such as self-representation or entertainment, older adults may be more motivated to engage in the virtual world for social reasons, such as maintaining interactions with their family, friends, or their community and receiving social support [32,33], which can be related to cognitive functioning [34].

For the aforementioned reasons, this study examined whether engagement in diverse internet-based social activities is positively related to concurrent and later cognitive functioning in middle-aged and older US adults beyond face-to-face social activities and whether this relationship varies by age. We hypothesized that internet-based social activity participation would be associated with high levels of concurrent cognitive functioning and slow cognitive decline across time, particularly in episodic memory. We also hypothesized that the relationship between internet-based social activity participation and cognitive functioning would be more pronounced in older adults. Data are from the Health and Retirement Study (HRS), a large national survey of adults aged 50 years and older. Information about technology use was first collected during the COVID-19 pandemic, a period when face-to-face interactions were limited and technology use among older adults increased [35].

## Methods

### Participants

The HRS is an ongoing project since 1992 that examines sociodemographic and psychological factors as well as health status to assess the well-being of US adults aged 50 years and older. The sample was recruited at the household financial unit level through a multistage area probability sampling method and required participants to complete an in-person interview and a telephone-administered short cognitive battery every 2 years [36]. From 2006 onward, a random 50% of participants were invited to complete a psychosocial survey returned to the researchers by mail. For the cross-sectional analysis, we used data from 2020 (n=3650), when survey questions about activity engagement in both internet-based and offline (face-to-face) settings in the self-administered psychosocial survey were first included. Data were collected from March 2020 through May 2021, amid the COVID-19 pandemic. For the longitudinal analysis, we included 2034 participants who completed cognitive assessments both in 2020 (wave 1) and 2022 (wave 2; collected from March 2022 through September 2023).

### Measures

#### Cognitive Functioning

Cognitive functioning was assessed using the modified Telephone Interview for Cognitive Status [37,38]. The Telephone Interview for Cognitive Status measures episodic memory, working memory, and attention and processing speed with the following tasks: the immediate and delayed recall of a set of 10 words (episodic memory; range of 0‐20), serial 7 subtraction (working memory; range of 0‐5), and backward counting (attention and processing speed; range of 0‐2). The scores for these 3 domains were summed together, with potential total scores ranging from 0 to 27, a method consistent with previous studies assessing overall cognitive functioningusing data from the HRS [39,40]. To address potential bias from missing cognitive data, the HRS imputed missing values in the 2020 dataset used in this study, using demographic factors (eg, birth year, years of education), wave-specific demographics (eg, age), economic status (eg, income), health factors (eg, chronic conditions, visual impairment), physical functioning (eg, instrumental activities of daily living [IADLs]), and prior cognitive functioning [41].

#### Internet-Based Social Activity

Internet-based social activity included the following 7 items: taking or sharing photos and videos; sending or receiving instant messages, text messages, or emails; writing or reading blogs, reviews, ratings, or comments on the internet; accessing a social network site like Facebook, Twitter, or Instagram; using other social media such as Linkedin to network with people; using WhatsApp, Snapchat, or similar apps to network with people; or connecting face-to-face with family and friends using an app (such as FaceTime or Skype). To calculate internet-based social activity scores, we first created binary variables indicating whether the participants reported having participated in such activity at least once a month or more (1=engaged at least once a month or more, 0=engaged less than once a month) and then summed these 7 binary composite variables, using a method consistent with the previous studies examining social activity variety [6,42]. Higher scores indicate more diverse internet-based social activities, with a possible range of 0 to 7.

#### Face-to-Face Social Activity

Face-to-face social activity included 11 items. Five items, indicative of socially oriented activities from prior research [43], included doing volunteer work with children or young people; doing any other volunteer or charity work; taking an educational or training course; going to a sporting, social, or other club event; and attending meetings of nonreligious organizations. The other 6 items, representing social activities in the previous literature [44], included caring for a sick or disabled adult; attending a religious service; doing activities with grandchildren, nieces, nephews, or neighborhood children; meeting up with children not living with the respondent; doing activities with other family members not living with the respondent; or meeting friends. We calculated the face-to-face social activity scores using the same method for internet-based social activity, with a possible range of 0 to 11. Higher scores indicate more diverse face-to-face social activities, following the existing studies on social activity variety [6,42].

#### Demographic and Health-Related Variables

We included the following demographic covariates in the model based on the previous studies on activity engagement [6,45]: age, sex (0=female, 1=male), race (0=racial and ethnic minority groups, 1=White), years of education (possible range: 0‐17), working status (0=not working, 1=working), marital status (0=not married or partnered, 1=married). To capture subjective economic status, we included a question about participants’ satisfaction with their financial situation (1=not at all satisfied, 5=completely satisfied). Two physical health-related factors were also included, one of which was IADLs (eg, difficulty with making phone calls, managing money, taking medications, shopping for groceries, preparing a hot meal), along with the number of chronic conditions (eg, hypertension, diabetes, cancer, lung disease, heart disease, stroke, arthritis). We also included an 8-item short form adapted from the 20-item Center for Epidemiologic Studies-Depression scale to measure depressive symptoms [46]. Participants indicated whether they had experienced any of the following during the past week with a yes (1) or no (0): felt depressed, felt that everything they did was an effort, slept restlessly, were happy (reverse coded), felt lonely, enjoyed life (reverse coded), felt sad, and could not get going.

### Data Analysis

Using SAS 9.4, we conducted 2 separate linear regression models for overall cognitive functioning. Model 1 included only internet-based social activity and covariates as predictors. Model 2 incorporated face-to-face social activity in Model 1 to determine whether the effects were held after a measure of social activity without technology was included. Then, we ran a regression to examine whether internet-based social activity was related to a change in cognitive functioning, after adjusting for baseline cognitive functioning and face-to-face social activity and covariates, using cognitive functioning in 2022 as the outcome and including cognitive functioning in 2020 along with the covariates and social activity.

We also ran regression models to examine the relationship between internet-based social activity and each subdomain of cognitive functioning to determine whether effects varied across these domains. In addition, we explored whether age interacted with internet-based social activity in its association with cognitive functioning, reasoning that a 2-year change in cognitive functioning may be more likely to occur in older individuals. We also explored whether age was perhaps more sensitive to influences on cognitive functioning.

### Ethical Considerations

This study used publicly available, deidentified secondary data from the HRS, which was approved by the University of Michigan Institutional Review Board and sponsored by the National Institute on Aging (NIA-U01AG009740). Therefore, institutional review board approval was not required.

## Results

### Characteristics of the Participants and Their Internet-Based Social Activity Participation

Participants in the cross-sectional sample (N=3650) were aged between 50 and 99 years (mean 67.68, SD 9.77) and most were female (2174/3650, 59.6%), White (2679/3650, 73.4%), not working (2579/3650, 70.7%), and married or partnered (2100/3650, 57.5%). Most participants were high school graduates with an average of 13.50 years of education and were, on average, satisfied with their current financial situation. Most reported good health, with a low average IADL score and few chronic conditions (3132/3650, 85.8%, had an IADL score of 0). On average, they engaged in 3.35 different types of face-to-face social activities and 3.50 types of internet-based social activities. Cognitive functioning ranged from 0 to 27 with an average score of 16.24 and an SD of 4.35. Table 1 presents additional demographic and health-related characteristics, along with details on social activities and cognitive functioning for the longitudinal sample and dropouts.

**Table 1.** Descriptive statistics in demographic factors, internet-based and offline social activity, and cognitive functioning.

	Cross-sectional sample (N=3650)	Longitudinal sample (n=2034)	Dropouts (n=1616)	Attrition analysis	
				*t* test or *χ*^*2*^ (*df*)	*P* value
Age at wave 1 (years), mean (SD)	68.68 (9.77)	67.85 (9.63)	69.71 (9.86)	−5.71 (3426.4)^a^	<.001
Sex (female), n (%)	2174 (59.6)	1220 (60)	954 (59)	0.33 (1)^b^	.56
Race (White), n (%)	2679 (73.4)	1382 (67.9)	1297 (80.3)	69.95 (1)^b^	<.001
Marital status (married), n (%)	2100 (57.5)	1147 (56.4)	953 (59)	2.46 (1)^b^	.12
Working status (currently working), n (%)	1071 (29.3)	623 (30.6)	448 (27.7)	3.67 (1)^b^	.06
Years of education, mean (SD)	13.50 (2.79)	13.23 (2.82)	13.85 (2.72)	−6.68 (3513.7)^a^	<.001
Satisfaction with financial situation, mean (SD)	3.54 (1.09)	3.48 (1.10)	3.60 (1.07)	−3.34 (3500.7)^a^	<.001
Number of chronic conditions, mean (SD)	2.09 (1.36)	2.07 (1.31)	2.12 (1.41)	−1.11 (3648)^a^	.27
Instrumental activities of daily living, mean (SD)	0.23 (0.65)	0.22 (0.62)	0.24 (0.69)	−0.92 (3648)^a^	.36
Depressive symptoms, mean (SD)	1.31 (1.92)	1.35 (1.93)	1.28 (1.90)	1.10 (3485.3)^a^	.27
Face-to-face social activity, mean (SD)	3.35 (2.05)	3.36 (2.04)	3.33 (2.05)	0.40 (3452.7)^a^	.69
Internet-based social activity, mean (SD)	3.50 (2.00)	3.38 (2.09)	3.64 (1.88)	3.81 (3648)^a^	<.001
Cognitive functioning, mean (SD)	16.24 (4.35)	16.18 (4.31)	16.32 (4.40)	−0.99 (3430.3)^a^	.32

a *t* test.

b Chi-square test.

The 7 types of internet-based social activities varied in their frequency, with the most frequent being sending or receiving instant messages, text messages, or emails (3049/3650, 83.5%) and the least being using social media platforms such as LinkedIn (611/3650, 16.7%) (Table 2).

**Table 2.** Number of participants engaging in various internet-based social activities.

Internet-based social activity	Participants (N=3650), n (%)
Sending or receiving instant messages, text messages, or emails	3049 (83.5)
Taking or sharing photos and videos	2360 (64.7)
Accessing a social network site like Facebook, Twitter, or Instagram	2323 (63.6)
Writing or reading blogs, reviews, ratings, or comments on the internet	2011 (55.1)
Connecting face-to-face with family and friends using an app such as FaceTime or Skype	1695 (46.4)
Using WhatsApp, Snapchat, or similar apps to network with people	713 (19.5)
Using other social media such as LinkedIn to network with people	611 (16.7)

### Internet-Based Social Activity Participation and Concurrent Cognitive Functioning

Table 3 presents the results from our regression models testing our hypothesis that a greater variety of internet-based social activity use would be related to higher concurrent cognitive functioning. As indicated in Model 1, those who engaged in more diverse internet-based social activities had better overall cognitive functioning after adjusting for demographic and health-related factors (*b*=0.46, SE 0.07, *P*<.001). Results remained significant when we added face-to-face social activity participation (*b*=0.44, SE 0.07, *P*<.001; Model 2).

**Table 3.** Cross-sectional associations of internet-based social activity at W1^a^ with cognitive functioning at W1 (n=3650). Age, internet-based social activity participation, and face-to-face social activity participation were standardized.

Parameter	Model 1		Model 2	
	*b* (SE)	*P* value	*b* (SE)	*P* value
Intercept	11.09 (0.45)	<.001	11.13 (0.45)	<.001
Age	−0.75 (0.08)	<.001	−0.75 (0.08)	<.001
Men (vs women)	0.71 (0.13)	<.001	0.70 (0.13)	<.001
White (vs racial and ethnic minority groups)	−1.20 (0.15)	<.001	−1.21 (0.15)	<.001
Married (vs not married or partnered)	0.23 (0.14)	.09	0.22 (0.14)	.10
Currently working (vs not working)	0.29 (0.16)	.06	0.28 (0.16)	.08
Education	0.36 (0.02)	<.001	0.36 (0.02)	<.001
Satisfaction with financial situation	0.17 (0.06)	.008	0.16 (0.06)	.01
Instrumental activities of daily living	−1.05 (0.10)	<.001	−1.04 (0.10)	<.001
Number of chronic conditions	−0.06 (0.05)	.27	−0.06 (0.05)	.27
Depressive symptoms	−0.20 (0.04)	<.001	−0.20 (0.04)	<.001
Internet-based social activity at W1^b^	0.46 (0.07)	<.001	0.44 (0.07)	<.001
Face-to-face social activity at W1^c^	—^d^	—	0.11 (0.07)	.09
Adjusted *R^2^*	0.24	—	0.24	—
*F* statistic	106.59	<.001	98.00	<.001

a W1: wave 1.

b Online social activity participation at W1 remained significant when we excluded the item “taking or sharing photos and videos” (*b*=0.39*,* SE 0.07; *P*<.001).

c When examining the relationship between face-to-face social activity participation and cognitive functioning, excluding internet-based participation, face-to-face social activity participation was related to high levels of cognitive functioning (*b*=0.18, SE 0.06; *P*<.001).

d Not applicable.

### Internet-Based Social Activity Participation and Later Cognitive Functioning

We then examined our prediction that greater variety in internet-based social activity participation in 2020 would be related to a slower decline in cognitive functioning over 2 years. Results from our longitudinal regression analysis revealed that those who engaged in more diverse internet-based social activities had higher cognitive functioning 2 years later, after adjusting for demographic and health-related factors as well as for cognitive functioning at baseline (*b*=0.36, SE 0.09, *P*<.001; Model 1 in Table 4). Results remained significant when we added face-to-face social activity participation (*b*=0.35, SE 0.09, *P*<.001; Model 2 in Table 4).

**Table 4.** Longitudinal associations of internet-based social activity at W1^a^ with cognitive functioning at W2^b^ (n=2034). Age, internet-based social participation, and face-to-face social activity participation were standardized.

Parameter	Model 1		Model 2	
	*b* (SE)	*P* value	*b* (SE)	*P* value
Intercept	5.67 (0.56)	<.001	5.68 (0.56)	<.001
Age	−0.72 (0.09)	<.001	−0.72 (0.09)	<.001
Men (vs women)	−0.06 (0.16)	.70	−0.06 (0.16)	.69
White (vs racial and ethnic minority groups)	−0.54 (0.16)	.001	−0.54 (0.16)	.001
Married (vs not married or partnered)	0.11 (0.16)	.48	0.11 (0.16)	.49
Currently working (vs not working)	0.34 (0.18)	.06	0.34 (0.18)	.06
Education	0.19 (0.03)	<.001	0.19 (0.03)	<.001
Satisfaction with financial situation	0.03 (0.07)	.69	0.03 (0.07)	.71
Instrumental activities of daily living	−0.18 (0.13)	.16	−0.18 (0.13)	.17
Number of chronic conditions	−0.09 (0.06)	.13	−0.09 (0.06)	.13
Depressive symptoms	−0.08 (0.04)	.06	−0.08 (0.04)	.06
Cognitive functioning at W1	0.49 (0.02)	<.001	0.49 (0.02)	<.001
Internet-based social activity at W1	0.36 (0.09)	<.001	0.35 (0.09)	<.001
Face-to-face social activity at W1^c^	—^d^	—	0.03 (0.08)	.71
Adjusted *R^2^*	0.44	—	0.44	—
*F* statistic	136.65	<.001	126.10	<.001

a W1: wave 1.

b W2: wave 2.

c When examining the relationship between face-to-face social activity participation and cognitive functioning, excluding internet-based participation, face-to-face social activity participation was not associated with change in cognitive functioning across time (*b*=0.08, SE 0.07; *P*=.27).

d Not applicable.

### Additional Exploratory Analyses

When we examined the longitudinal association of internet-based social activity participation with changes in subdomains of cognitive functioning, those who participated in more diverse internet-based social activities had better later episodic memory (*b*=0.35, SE 0.08; *P*<.001), but not working memory or attention and processing speed, after adjusting for the baseline cognitive functioning (Table 5). In addition, we explored possible age differences in the association of social activity participation with later cognitive functioning, but the interaction effect of age and internet-based social activity participation was not significant for overall concurrent cognitive functioning (Table 6).

**Table 5.** Longitudinal associations of internet-based social activity at W1^a^ with subdomains of cognitive functioning at W2^b^ (n=2034). Age, internet-based social participation, and face-to-face social activity participation were standardized.

Parameter	Episodic memory		Working memory		Attention and processing speed	
	*b* (SE)	*P* value	*b* (SE)	P value	*b* (SE)	*P* value
Intercept	4.11 (0.47)	<.001	1.15 (0.20)	<.001	1.39 (0.08)	<.001
Age	−0.64 (0.08)	<.001	−0.10 (0.03)	.003	−0.03 (0.01)	.03
Men (vs women)	0.22 (0.14)	.11	−0.22 (0.06)	<.001	0.00 (0.02)	.90
White (vs racial and ethnic minority groups)	−0.31 (0.14)	.03	−0.24 (0.06)	<.001	−0.03 (0.02)	.16
Married (vs not married or partnered)	0.06 (0.14)	.67	0.06 (0.06)	.34	0.00 (0.02)	.96
Currently working (vs not working)	0.33 (0.16)	.03	0.02 (0.07)	.77	−0.01 (0.03)	.75
Education	0.13 (0.02)	<.001	0.06 (0.01)	<.001	0.02 (0.00)	<.001
Satisfaction with financial situation	0.01 (0.06)	.87	0.03 (0.03)	.25	0.00 (0.01)	.78
Instrumental activities of daily living	−0.15 (0.11)	.18	−0.05 (0.05)	.28	−0.01 (0.02)	.45
Number of chronic conditions	−0.08 (0.05)	.15	−0.03 (0.02)	.23	0.01 (0.01)	.29
Depressive symptoms	−0.07 (0.04)	.07	−0.01 (0.02)	.44	−0.01 (0.01)	.08
Episodic memory, working memory, or attention and processing speed	0.42 (0.02)	<.001	0.52 (0.02)	<.001	0.16 (0.02)	<.001
Face-to-face social activity at W1	0.05 (0.07)	.42	−0.01 (0.03)	.66	−0.01 (0.01)	.18
Internet-based social activity at W1	0.35 (0.08)	<.001	0.03 (0.03)	.30	−0.00 (0.01)	.70
Adjusted *R^2^*	0.38	—^c^	0.37	—	0.04	—
*F* statistic	96.49	<.001	93.15	<.001	7.09	<.001

a W1: wave 1.

b W2: wave 2.

c Not applicable.

**Table 6.** Interaction effect of age and internet-based social activity participation with cognitive functioning at wave 2 (n=2034). Age, internet-based social participation, and face-to-face social activity participation were standardized.

Parameter	Model 1		Model 2	
	*b* (SE)	*P* value	*b* (SE)	*P* value
Intercept	5.65 (0.56)	<.001	5.65 (0.56)	<.001
Age	−0.74 (0.10)	<.001	−0.74 (0.10)	<.001
Men (vs women)	−0.07 (0.16)	.67	−0.07 (0.16)	.67
White (vs racial and ethnic minority groups)	−0.53 (0.16)	.001	−0.53 (0.16)	.001
Married (vs not married or partnered)	0.11 (0.16)	.48	0.11 (0.16)	.49
Currently working (vs not working)	0.32 (0.18)	.07	0.34 (0.18)	.07
Education	0.19 (0.03)	<.001	0.19 (0.03)	<.001
Satisfaction with financial situation	0.03 (0.07)	.72	0.02 (0.07)	.74
Instrumental activities of daily living	−0.18 (0.13)	.16	−0.18 (0.13)	.17
Number of chronic conditions	−0.09 (0.06)	.13	−0.09 (0.06)	.13
Depressive symptoms	−0.08 (0.04)	.06	−0.08 (0.04)	.06
Cognitive functioning at W1^a^	0.50 (0.02)	<.001	0.49 (0.02)	<.001
Internet-based social activity at W1	0.35 (0.09)	<.001	0.35 (0.09)	<.001
Face-to-face social activity at W1	—^b^	—	0.03 (0.08)	.71
Age × internet-based social activity participation	−0.06 (0.07)	.41	−0.06 (0.07)	.41
Adjusted *R^2^*	0.44	—	0.44	—
*F* statistic	126.17	<.001	117.12	<.001

a W1: wave 1.

b Not applicable.

## Discussion

Building on the importance of social activity in the real world, we examined the impact of a variety of social activities in virtual worlds on cognitive health. We found that greater engagement in internet-based social activities was associated with high levels of concurrent and later cognitive functioning, particularly in episodic memory, even after adjusting for face-to-face social activity, a relationship that did not vary by age.

### Internet-Based Social Activity Participation With Cognitive Functioning

People have raised concerns that virtual social activities may not provide the same mental stimulation as face-to-face interactions, due to factors such as reduced social cues from the absence of facial expressions or voice tones [46]. The implication of these concerns is that less social stimuli would provide less cognitive stimulation. Yet, research finds that virtual communication offers a variety of different types of cues and multiple sensory stimulation [20]. For example, video-based communications involve similar verbal and nonverbal cues to face-to-face social activities [47]. Dynamic animations or videos also provide visual and auditory cues [48,49], which may enhance one’s engagement with the environment. In addition to potential cognitive stimulation from communicating with others in these virtual worlds, internet-based social activity includes other tasks involving cognitive processing, such as recollecting, retrieving, and reminding functions [30]. Moreover, internet-based social activity participation may be a channel to exchange social support, which further relates to cognitive functioning [7]. Platforms like Snapchat or Instagram enable sharing moments across generations, while blogging fosters a sense of belonging and social support by connecting individuals with shared interests [50,51], all of which can be potential psychological mechanisms for cognitive benefits and may be reasons why engaging in virtual social activities was associated with high levels of both concurrent and later cognitive functioning in this study.

When examining the subdomains of cognitive functioning, engagement in diverse internet-based social activities was only related to episodic memory. Engagement in social activities in virtual worlds may facilitate learning and remembering new information, which has been related to better episodic memory in another study [52] and is consistent with our findings. On the other hand, activities included in this study, such as posting, messaging, and sharing photos, have not been associated with executive functioning in previous studies [30], similar to our null effects within this subdomain. A recent study, however, suggests an indirect relationship between internet-based social activity participation and executive functioning, as seen in individuals who receive social support through sustained use of internet-based social media [34]. We do not have the data to examine this indirect relationship, but future studies should examine the possibility of indirect effects of virtual social activity participation on executive functioning.

The relationship between internet-based social activity participation and cognitive functioning did not vary by age. Previous research raised concerns that older adults may derive fewer benefits from internet-based social activities because they are likely to have potential physical limitations restricting engagement in technology use in social interactions [53]. Yet, our results indicate that the cognitive benefits of internet-based social activity participation were consistent across age even without adjusting for health-related factors. In addition, the potential mechanisms that may link participation in internet-based social activities and cognitive functioning may be comparable across ages, as internet users of all ages tend to participate in various social networks [54].

### Associations of Face-to-Face Social Activity Participation With Cognitive Functioning

Face-to-face social activity participation was also related to concurrent cognitive functioning, but not to later cognitive functioning, when internet-based participation was excluded from the model. Our results contrast previous findings indicating cognitive benefits of face-to-face social activities [8]. One potential reason why we failed to find a difference is that the effect size of the relationship between face-to-face social activity participation and cognitive functioning may be very small. Another reason may be that people who may experience cognitive decline or other cognitive issues may receive more social support from others and thus obscure the association. Finally, these data were collected during a time when face-to-face interactions were more limited than usual. Social activities outside the home, such as clubs, educational courses, or volunteer work, were strictly restricted during the COVID-19 pandemic. This may have led to a limited engagement in face-to-face social activities regardless of individuals’ interests or motivations for engaging in them and may have contributed to the weaker association observed with cognitive functioning.

Another consideration is that cognitive health benefits of face-to-face social activity may be especially notable when individuals are involved in these activities consistently over an extended period of time [6]. Studies have supported the importance of sustained social activity participation by demonstrating that the relationship between face-to-face social activity and cognitive functioning was stronger when examining the participation across time rather than participation at the baseline [55]. We did not measure how long participants had been engaging in each of these activities.

Importantly, this study focused on cognitive functioning, one aspect of health, among older adults. Our findings suggest that virtual social activities, as opposed to face-to-face activities, were related to a slower decline in cognitive functioning over 2 years; however, other aspects of health may have a different pattern of results. For example, several studies on the health benefits of internet-based and face-to-face social activity participation have found that only face-to-face interactions are related to better emotional well-being [56]. In addition, several studies find that social interactions via technology such as emailing or texting cannot replace face-to-face interactions in combating loneliness [57], emphasizing the significance of face-to-face social interactions for other health outcomes, such as emotional health.

### Limitations and Future Directions

This study has limitations that can be addressed in future studies. Internet-based social activities used in this study may not encompass all social activities in the virtual world. Further studies could examine a broader range of such activities, such as playing interactive computer games. Moreover, the item “taking or sharing photos and videos” may capture 2 distinct activities: taking photos or videos and sharing them. Since sharing involves social interaction, those who only took photos may not be engaging socially. We examined whether excluding this item from internet-based social activity impacts its relationship with cognitive functioning, and the results remained consistent. Although we used items with social components, future studies could explore which internet-based activities necessarily involve social interaction and how they relate to cognitive functioning. In addition, we used data that were collected during the COVID-19 pandemic, and as we mentioned, engagement in various internet-based social activities may have health benefits when in-person interactions are limited—benefits that may not appear when people are less socially restricted. Future studies could examine the relationship between internet-based social activity participation and cognitive functioning under other circumstances. Lastly, other psychological or sociodemographic factors, such as motivations for learning, could yield different patterns of the relationship between engagement in a variety of internet-based social activities and cognitive functioning. Further studies could explore this relationship across different backgrounds, such as in individuals with different levels of motivations or self-control.

### Conclusion

Social activities through internet-based platforms, such as sharing moments or communicating with others, were related to higher levels of concurrent and future cognitive functioning in late adulthood when examined during the COVID-19 pandemic. This study contributes to the existing findings on the importance of social activity participation by promoting the role of engagement in diverse social activities in internet-based settings. Given that middle-aged and older adults are highly motivated to interact with their friends and family members, they may be more likely to use and benefit from technology that provides them with a way to maintain social connections.

## Abbreviations

HRS: Health and Retirement Study
IADL: instrumental activity of daily living
